# Supplementary material for: Quantitative Evaluation of Interleukin-4 by Immunowall Devices Made of Gelatin Methacryloyl Hydrogel
Source: Molecules. 2023 Jun 8;28(12):4635. doi: 10.3390/molecules28124635 (PMC10304311; doi:10.3390/molecules28124635)
Supplement: Supplementary file 1 [file molecules-28-04635-s001.zip › molecules-2414899-supplementary.pdf]

Quantitative Evaluation of Interleukin-4 by Immunowall Devices Made of Gelatin Methacryloyl Hydrogel

Yuto Banno, Takuma Nomiyama, Shoma Okuno, Sachiko Ide and Noritada Kaji *

Department of Applied Chemistry, Graduate School of Engineering, Kyushu University, 744 Motooka, Nishi-ku, Fukuoka 819-0395, Japan
* Correspondence: kaji@cstf.kyushu-u.ac.jp; Tel.: +81-92-802-2883

Keywords: Immunoassay, Lab on a Chip, Interleukin, Gelatin Methacryloyl hydrogel

Supporting information

Table S1. Experimental conditions of IL-4 immunoassay to address the small fluorescence at no IL-4 (antigen) condition.

	Biotin-labeled primary antibody	IL-4 (antigen)	Secondary antibody	Fluorescent-labeled tertiary antibody
a	—	—	50 µg/mL	50 µg/mL
b	50 µg/mL	—	—	50 µg/mL
c	—	—	—	50 µg/mL
d	50 µg/mL	—	50 µg/mL	50 µg/mL
e	50 µg/mL	—	50 µg/mL	—
f	—	—	—	—

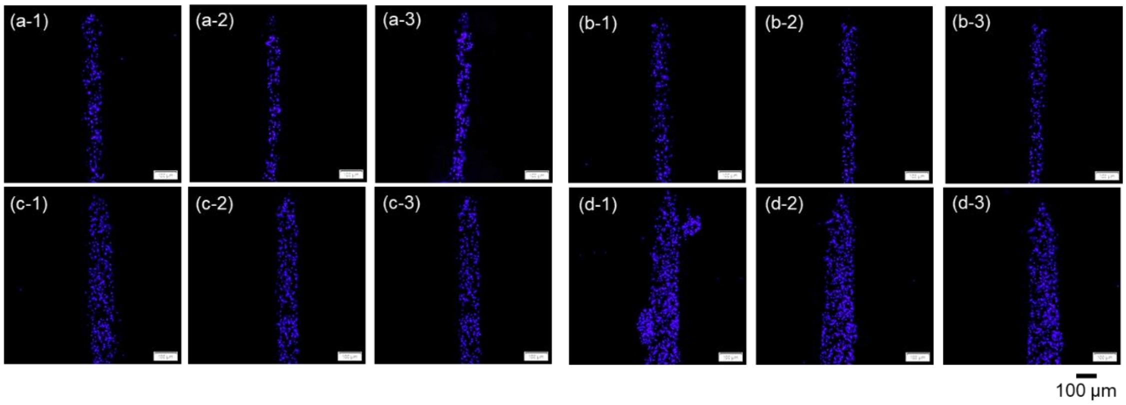

Figure S1. Fluorescence microscope images of 1.0 µm-fluorescence beads premixed GelMA hydrogel under different UV irradiation time and washing repetition count. The UV irradiation time was (a) 50

s, (b) 100 s, (c) 200 s, and (d) 300 s. The sub numbers following the alphabets (*) indicate the different repetition number of washing process, (*-1) 1, (*-2) 5, (*-3) 10 times washing. Longer exposure time showed wider wall but the captured fluorescence beads did not leak from the UV cured wall regardless of the wall width.

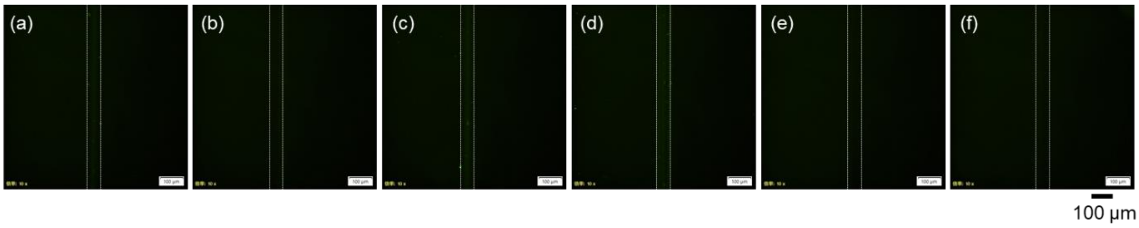

Figure S2. Fluorescence microscope images of the walls after immunoassay under conditions of various reagent combinations as shown in Table S1. The white dotted lines indicate the edges of the wall just to guide the eye. Condition (a), (c), and (d) showed slight fluorescence compared with the background.
